# Abdominal allergic reaction syndrome: a tentative definition

**DOI:** 10.3389/fmed.2026.1929771

**Published:** 2026-10-05

**Authors:** Meiyi You, Xinglan Liao, Xueqi Li, Lan Guan, Zhaoxing Tian

**Affiliations:** Department of Emergency Medicine, Beijing Jishuitan Hospital, Capital Medical University, Beijing, China

**Keywords:** abdominal pain, acute abdomen, corticosteroid, diagnostic framework, hypersensitivity

## Abstract

**Background:**

Some patients with acute abdominal pain present with imaging findings suggestive of severe intestinal pathology, yet they remain hemodynamically stable and respond to corticosteroids rather than surgery. Recognizing this pattern early can prevent unnecessary laparotomy.

**Cases:**

Three female patients (aged 91, 78, and 57 years) admitted to the emergency department presented with acute abdominal pain and diffuse bowel wall edema on a CT scan. Despite alarming imaging results, all three patients remained hemodynamically stable. The symptoms improved rapidly with intravenous methylprednisolone, and all patients were discharged without surgery.

**Conclusions:**

We propose Abdominal Allergic Reaction Syndrome (AARS) as a preliminary recognition framework for this clinico-radiological pattern. AARS is not intended to replace specific diagnoses such as immunoglobulin A (IgA) vasculitis or lupus enteritis. Rather, it serves as an early identifier at a stage when the underlying etiology remains unclear. The core features (hemodynamic stability and clinico-radiological mismatch, supported by normal lactate levels) may help emergency physicians avoid unnecessary surgery while proceeding with the diagnostic workup. The term “allergic” is used here in its broader immunological sense (encompassing all type I–IV hypersensitivity reactions) and is not restricted to IgE-mediated reactions.

## Introduction

Acute abdominal pain accounts for approximately 10% of emergency department visits globally (1). Current guidelines emphasize early cross-sectional imaging to identify structural anomalies requiring urgent surgery (2, 3), and emergency laparotomy itself carries risks for substantial morbidity and mortality, including a non-negligible rate of non-therapeutic procedures (4–6); in one series of 27 negative laparotomies performed for acute abdomen, three patients were ultimately found to have an autoimmune disease (5). A subset of patients presents a particular dilemma: the imaging results show severe diffuse bowel wall edema, ascites, and even portal venous gas, yet vital signs and lactate levels remain reassuringly normal.

Several immune-mediated conditions can produce this pattern. Eosinophilic gastroenteritis (EGE) (7), immunoglobulin A (IgA) vasculitis (8), and lupus enteritis (9) cause immune-driven intestinal inflammation that mimics a surgical abdomen on imaging but responds to corticosteroids. These are distinct diseases with different pathomechanisms, and each already has its own diagnostic criteria. However, the practical challenge emerges earlier: when a patient arrives with acute abdominal pain and the specific etiology is not yet established, how should the emergency physician decide between surgical exploration and a trial of conservative management?

We propose the concept of Abdominal Allergic Reaction Syndrome (AARS) to address this clinical gap. It is not a new disease entity but a transitional recognition framework for the early, etiology-undefined stage of presentation. Its value lies in the interval between initial presentation and definitive diagnosis: identifying the pattern of hemodynamic stability and clinico-radiological mismatch, supported by normal lactate levels, may help clinicians identify patients who do not require surgery and spare them from unnecessary surgical procedures. Recognition of this pattern is not, by itself, an indication for corticosteroid therapy; when an immune-mediated etiology is reasonably suspected and competing infectious and surgical causes have been appropriately evaluated, corticosteroids may be considered on an individual basis while the targeted diagnostic workup proceeds.

## Case presentations

### Case 1

A 91-year-old woman with well-controlled type 2 diabetes mellitus and essential hypertension presented 12 h after the onset of periumbilical pain, nausea, and dizziness. She was afebrile (36.1 °C), hemodynamically stable (heart rate, 89 beats/min; blood pressure, 132/65 mmHg), and alert. The white blood cell (WBC) count was 5.93 × 10^9^ cells/L (64% neutrophils) with a normal eosinophil count, and blood biomarker levels are given as follows: lactate, 1.1 mmol/L; C-reactive protein (CRP), 10.9 mg/L; and D-dimer, 1.18 mg/L fibrinogen equivalent units (FEU). Contrast-enhanced CT showed diffuse small bowel wall edema, mild bowel dilation, small-volume pelvic free fluid (Figure 1A), and a significant amount of intrahepatic portal venous gas (Figure 1B). The patient was intensively monitored in the emergency room, with abdominal signs assessed every hour, and was prepared for immediate surgery in the event of any deterioration occurring. No mesenteric vessel occlusion or perforation was identified. The patient received bowel rest, intravenous fluids, methylprednisolone 80 mg on day 1 and then 40 mg daily for days 2 and 3 (totally 3 days), and empirical imipenem. Autoimmune serology tests, immunoglobulin and complement measurements, and endoscopy with biopsy were not performed. Repeat CT at 48 h showed complete resolution of portal venous gas and marked reduction in bowel wall thickening (Figure 1C). Although antibiotics were used concurrently, the rapid improvement in radiological imaging results and symptom relief are more consistent with the effects of corticosteroids. The patient tolerated oral intake by day 5 and was discharged on day 6. At the 2-month follow-up, she remained asymptomatic.

**Figure 1:**
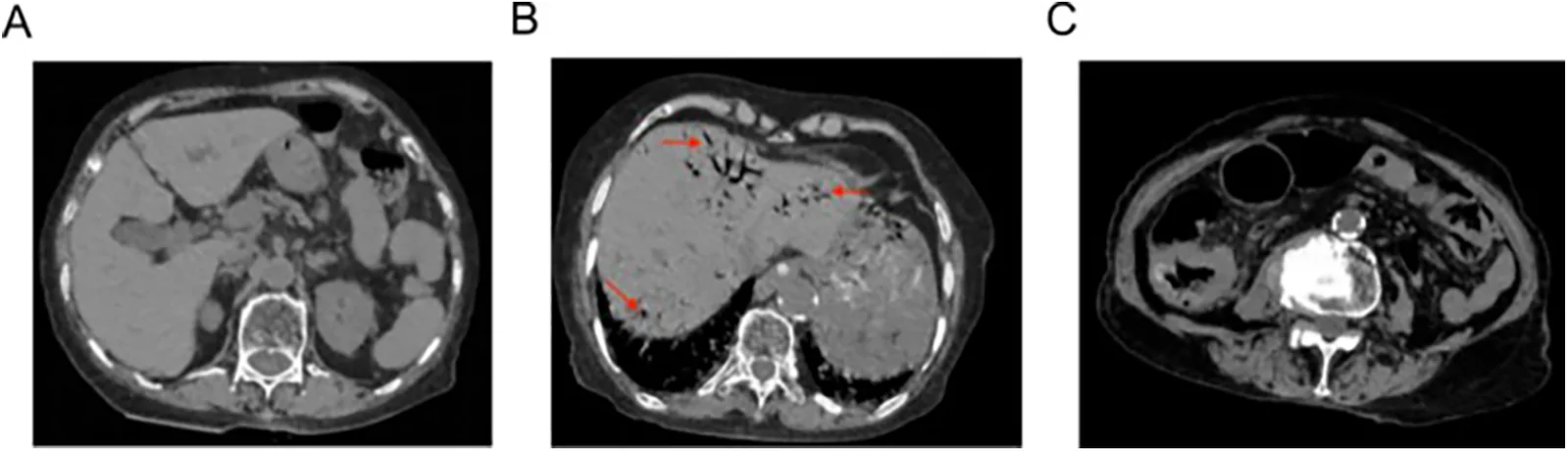
**(A)** Diffuse small bowel wall edema and pelvic free fluid. **(B)** Intrahepatic portal venous gas. **(C)** Resolution at 48 hours.

### Case 2

A 78-year-old woman with type 2 diabetes mellitus presented with 24 h of abdominal pain and one episode of melena. An examination showed only mild tenderness without peritoneal signs. The patient was afebrile and hemodynamically stable (heart rate, 97 beats/minute; blood pressure, 113/60 mmHg). The WBC count was 19.5 × 10^9^ cells/L (88% neutrophils) with a normal eosinophil count, the lactate level was 1.3 mmol/L, and the CRP level was 21.95 mg/L. CT revealed small bowel wall edema in the lower abdomen and pelvis, mesenteric fat stranding, and free fluid (Figures 2A, B). No active bleeding, thrombosis, or perforation was seen. Autoimmune serology tests, immunoglobulin and complement measurements, and endoscopy with biopsy were not performed. The patient received bowel rest, methylprednisolone 80 mg daily for 3 days and then 40 mg daily for 4 days, and empirical antibiotics. Pain resolved within 48 h. Follow-up CT at 2 weeks confirmed resolution of bowel wall edema (Figure 2C). She was discharged on day 8. At the 3-month follow-up, she remained asymptomatic.

**Figure 2:**
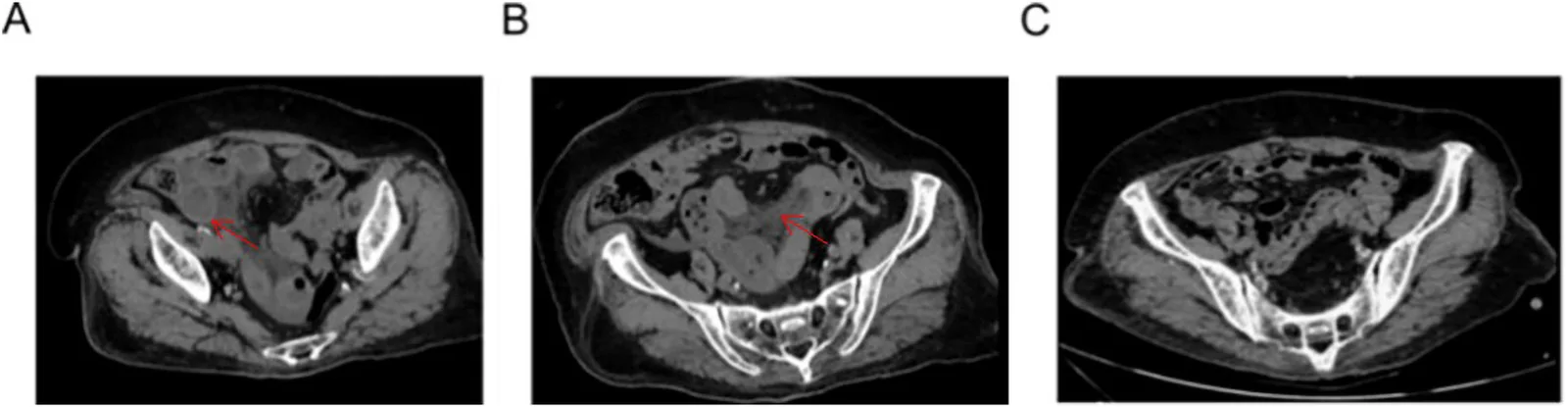
**(A,B)** Small bowel wall edema, mesenteric stranding, and free fluid. **(C)** Resolution at 2 weeks.

### Case 3

A 57-year-old woman with psoriasis and hearing–speech impairment developed periumbilical colicky pain 3 days after seafood consumption, along with bilateral limb edema, dizziness, and melena. She had been diagnosed with “acute gastroenteritis” elsewhere and received cefotaxime and metronidazole without improvement. On admission, she was afebrile and tachycardic (121 beats/min) and had a blood pressure of 143/94 mmHg, with bilateral limb edema. The WBC count was 24.6 × 10^9^ cells/L (92% neutrophils), the albumin level was 29 g/L, and the D-dimer level was 6.1 mg/L FEU. The lactate level on admission was not measured. Lower-limb Doppler and CT angiography excluded thrombosis. Abdominal CT showed focal jejunal wall thickening and ascites (Figures 3A, B).

**Figure 3:**
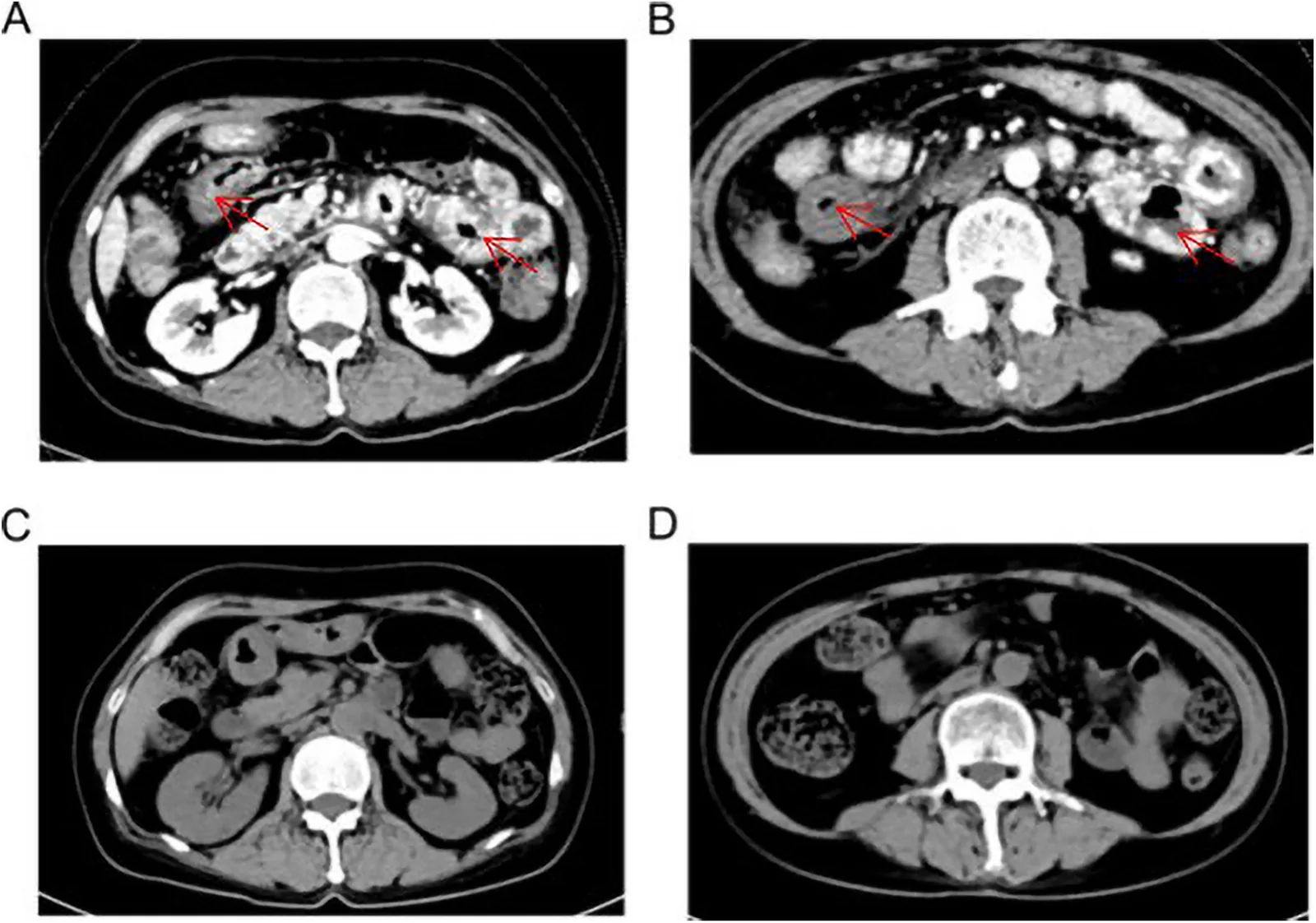
**(A,B)** Jejunal wall thickening and ascites. **(C,D)** Resolution after corticosteroid therapy.

Broad-spectrum antibiotics were initiated (ertapenem, piperacillin–tazobactam, and later escalated to tigecycline). Despite 5 days of antibiotic therapy, the symptoms worsened. Further testing showed an erythrocyte sedimentation rate of 25.0 mm/h, a C-reactive protein level of 8.83 mg/dL, a total complement result of 60.0 U/mL, an immunoglobulin M level of 0.418 g/L, and an immunoglobulin E level of 3.1 IU/mL. Antinuclear and anti–double-stranded DNA antibodies were negative, and the eosinophil percentage was 0. Recurrent melena lowered hemoglobin levels to 83 g/L. Gastroscopy and colonoscopy with biopsy showed neither vasculitis nor granulomas. When an immune-mediated etiology was reconsidered, intravenous methylprednisolone 80 mg daily was started. Abdominal pain alleviated within 12 hours. Methylprednisolone was continued (80 mg daily for 3 days and then 40 mg daily for 5 days). Limb edema resolved, inflammatory marker levels decreased, and a follow-up CT scan confirmed resolution of jejunal wall thickening and ascites (Figures 3C, D). At the 7-year follow-up, no recurrence occurred.

## Discussion

### AARS: what it is and what it is not

These three cases share a clinical pattern that we term AARS: acute abdominal pain with imaging evidence of bowel wall edema, yet preserved hemodynamics and normal lactate levels, followed by rapid response to corticosteroids. The key differential considerations in our cases (IgA vasculitis, eosinophilic gastroenteritis, ischemic bowel, and Crohn’s disease) were each considered and either supported or excluded based on clinical and laboratory features (10–13). The diagnostic evaluation of the three patients is summarized in Table 1.

**Table 1:** Clinical features, investigations, treatment, and outcomes of the three patients.

Feature	Case 1	Case 2	Case 3
Age/sex	91/F	78/F	57/F
Comorbidities	Type 2 diabetes mellitus; hypertension	Type 2 diabetes mellitus	Psoriasis; hearing–speech impairment
Onset to presentation	12 h	24 h	3 days
Vital signs	HR, 89 beats/min; BP, 132/65 mmHg; T, 36.1 °C	HR, 97 beats/min; BP. 113/60 mmHg	Afebrile; HR, 121 beats/min; BP, 143/94 mmHg; no signs of hypoperfusion
WBC (neutrophils, %)	5.93 × 10^9^/L (64%)	19.5 × 10^9^/L (88%)	24.6 × 10^9^/L (92%)
Eosinophils	Normal	Normal	0%
Lactate (mmol/L)	1.1	1.3	Not measured
CRP	10.9 mg/L	21.95 mg/L	88.3 mg/L
D-dimer (mg/L FEU)	1.18	Not performed	6.1
Main CT findings	Diffuse small bowel wall edema, pelvic free fluid, and portal venous gas	Small bowel wall edema (lower abdomen/pelvis), mesenteric fat stranding, and free fluid	Focal jejunal wall thickening and ascites
Vascular assessment	No mesenteric vessel occlusion or perforation on CTA	No active bleeding, thrombosis, or perforation on CTA	Lower-limb Doppler US and CT angiography: no thrombosis
Immunological tests	Not performed	Not performed	ANA and anti-dsDNA: negative; total complement, 60.0 U/mL; IgM, 0.418 g/L; IgE, 3.1 IU/mL; ESR, 25 mm/h
Endoscopy/biopsy	Not performed	Not performed	Gastroscopy and colonoscopy with biopsy: no vasculitis or granulomas
Other relevant findings	—	—	Melena; hemoglobin nadir, 83 g/L
Corticosteroid regimen	Methylprednisolone 80 mg on day 1 and then 40 mg daily for days 2 and 3	Methylprednisolone 80 mg daily for 3 days and then 40 mg daily for 4 days	Methylprednisolone 80 mg daily for 3 days and then 40 mg daily for 5 days
Antibiotics	Imipenem (empirical)	Empirical antibiotics	Ertapenem, piperacillin–tazobactam, and then tigecycline
Time to response	Marked radiological improvement within 48 h	48 h	12 h
Length of stay (days)	6	8	39
Follow-up	Asymptomatic at 2 months	Asymptomatic at 3 months	No recurrence at 7 years

HR, heart rate; BP, blood pressure; CTA, computed tomography angiography; US, ultrasound; ANA, antinuclear antibody; ESR, erythrocyte sedimentation rate.

It is worth noting that AARS does not replace these diagnoses. A patient eventually diagnosed with IgA vasculitis already has a well-defined disease entity with established criteria (10). AARS serves a different purpose: it addresses the window before that diagnosis is made. When a patient arrives in the emergency department with acute abdominal pain and imaging shows alarming bowel wall changes, the immediate question should not be “what specific disease is this?” Rather, it should be “does this patient need surgery now, or can I safely try conservative management?” AARS offers a preliminary answer to this question.

AARS is proposed as a clinical, syndrome-level diagnosis and not as a new disease entity. Its defining feature is a clinico-pathological process consistent with hypersensitivity, and the consistent effectiveness of antiallergic therapy constitutes a therapeutic (*ex juvantibus*) basis for the label: we observed a clinical phenomenon, and treatment directed toward hypersensitivity worked. Therefore, we do not speculate on the specific molecular mechanisms involved, which remain to be defined by future studies. The absence of eosinophilia or elevated IgE levels does not argue against a hypersensitivity process because many hypersensitivity reactions, particularly non–IgE-mediated ones, present without these findings. Similarly, tissue biopsy is neither suitable nor feasible for every patient, particularly older or frail emergency patients; the framework is designed precisely for the stage at which such information is not yet available.

### The clinico-radiological mismatch

The three cases share a striking dissociation: CT findings that would typically raise concerns for surgical pathology (bowel wall edema, ascites, and even portal venous gas) but hemodynamic stability and a lactate level below 2.0 mmol/L.

Portal venous gas has historically been considered ominous, but accumulating evidence shows that in the absence of bowel necrosis signs, conservative management can be successful (2). Similarly, diffuse bowel wall edema, although concerning, may reflect immune-mediated inflammation rather than irreversible ischemia. The crucial distinction in our cases was the combination of stable vitals, normal lactate levels, and the absence of pneumatosis intestinalis or free air—features that, taken together, suggested a reversible process rather than surgical bowel compromise.

The findings on lactate levels fit this interpretation. Hypersensitivity reactions can themselves progress to shock, and shock induced by any cause increases lactate levels; conversely, patients with comparable abdominal imaging results due to extensive infectious inflammation typically show markedly elevated lactate levels. In the case of our patients, lactate levels remained normal (1.1 and 1.3 mmol/L in cases 1 and 2, respectively), supporting, although not proving, a non-ischemic, non-infectious process. A normal serum lactate level does not, however, by itself exclude early mesenteric ischemia or other significant surgical pathologies; lactate is therefore a supporting feature of the framework rather than a mandatory criterion. The hemodynamic course points the same way: had the abdominal pathology implied by the imaging results been infectious or surgical in nature, hemodynamic compromise would be expected, yet our patients remained stable throughout. An isolated heart rate of 121 beats/minute with normal blood pressure and no signs of hypoperfusion, as in case 3, does not constitute hemodynamic instability. The speed of the response to corticosteroids reinforces the mismatch further: infectious diseases do not resolve within hours to days of steroid initiation.

### Immune-mediated conditions underlying AARS

Several established conditions can produce AARS.

IgA vasculitis (Henoch–Schönlein purpura) is a systemic IgA-mediated small vessel vasculitis with gastrointestinal involvement in over half of the patients (10). Intestinal leukocytoclastic vasculitis produces edema and pain that can be severe enough to mimic a surgical abdomen (8). Corticosteroids can rapidly reduce inflammatory edema and alleviate pain (14, 15).

EGE is characterized by eosinophilic infiltration of the gastrointestinal tract (11). Glucocorticoids are the preferred conservative treatment, with typical response within days (16–18). A diagnostic–therapeutic trial of corticosteroids can provide useful information even before biopsy results are available.

Lupus enteritis is an abdominal manifestation of systemic lupus erythematosus (SLE) and can be the sole presenting feature of the disease (19). Enhanced CT typically shows bowel wall thickening (“target sign”), mesenteric vascular congestion (“comb sign”), and ascites. Most patients respond rapidly to steroid therapy (20, 21).

Eosinophilic granulomatosis with polyangiitis (EGPA) can also involve the gastrointestinal tract (22). Notably, cases of EGPA-associated acute cholecystitis diagnosed preoperatively have improved with the use of glucocorticoids alone, whereas those diagnosed postoperatively had more complications (23), underscoring the value of preoperative recognition.

### Pathophysiological considerations

The favorable response to corticosteroids and the absence of systemic decompensation suggest that AARS involves localized immune-mediated microvascular dysfunction and intestinal wall edema, rather than progressive structural damage. Glucocorticoids suppress excessive release of inflammatory mediators, reduce capillary permeability, and relieve intestinal wall edema (24, 25). However, their precise mechanisms remain unclear. The allergic/immunological evidence in this case is insufficient, and the immunological nature of AARS still needs to be verified through prospective studies.

The treatment sequence supports this interpretation. In case 3, symptoms worsened during the 5 days of escalating broad-spectrum antibiotics and they improved within 12 h of corticosteroid initiation. Had the process been a conventional bacterial infection, effective antibiotics should have produced improvement, and corticosteroids would not be expected to resolve an uncontrolled infectious process and they might aggravate it. The rapid and sustained response to corticosteroids therefore argues for an immune-mediated, rather than, infectious mechanism. Nonetheless, we acknowledge that in cases 1 and 2, antibiotics were given concurrently, and therefore, the contribution of each treatment could not be fully separated; we read the steroid response as supportive and not confirmatory.

### AARS: a preliminary diagnostic framework

Based on our three cases and the literature on immune-mediated abdominal conditions, we propose a preliminary recognition framework:

#### Core features

1. Hemodynamic stability: normal blood pressure without progressive tachycardia; isolated tachycardia with normal blood pressure and no signs of hypoperfusion does not constitute instability.
2. Clinico-radiological mismatch: imaging shows diffuse bowel wall edema, ascites, or portal venous gas, but clinical severity does not match the imaging alarm.

#### Supporting features

1. Lactate level <2.0 mmol/L: an elevated lactate level is associated with bowel ischemia and adverse outcomes (26), but a normal level does not exclude early or intermittent ischemia; this feature is supportive and not mandatory.
2. No evidence of mechanical obstruction or active bleeding in a contrast-enhanced CT scan.
3. Inflammatory markers may be normal or elevated without corresponding organ dysfunction.
4. Poor response to empirical antibiotics alone.
5. Findings suggestive of immune-mediated conditions (eosinophilia, elevated levels of IgE and autoimmune markers, and a history of atopic or autoimmune disease); their absence does not exclude a hypersensitivity process.

#### Caveats

1. Hemodynamic instability, lactate level >2.0 mmol/L, pneumatosis intestinalis, or clear peritoneal signs should prompt surgical evaluation.
2. AARS is a transitional framework and not a final diagnosis; workup for specific etiologies must proceed in parallel.
3. These criteria are derived from three cases and require prospective validation.

### Management principles

All three patients received conservative management with corticosteroids, bowel rest, and intravenous fluids. In each case, the decision to start corticosteroids followed the same reasoning: empirical anti-infective therapy had failed or was deemed insufficient. The diffuse and uniform nature of the bowel involvement was considered more consistent with an immune-mediated process than with conventional bacterial infections, and after careful assessment, we adjudicated that corticosteroids were unlikely to cause adverse effects and were likely to benefit the patient. Symptom reduction occurred within 12–48 h. The general approach is given as follows:

1. Close hemodynamic monitoring,
2. Bowel rest with intravenous fluid resuscitation,
3. Corticosteroid therapy, with dose individualized to severity,
4. Serial monitoring of lactate and inflammatory marker levels,
5. Repeat imaging if the clinical status changes, and
6. Concurrent workup for the underlying etiology (autoimmune serology, allergy testing, and endoscopy/biopsy as appropriate).

The AARS framework does not preclude surgical consultation. Any clinical deterioration, new peritoneal signs, or failure to alleviate symptoms should trigger surgical reassessment. An equally important factor is dynamic re-evaluation: serial abdominal examinations, repeat lactate measurement, and repeat imaging whenever the clinical course is concerning. Such a disciplined reassessment allows the framework to avoid misdiagnosis without encouraging overtreatment; this balance (avoiding unnecessary laparotomy without delaying necessary surgery) is the purpose of this framework.

## Conclusions

AARS is proposed as a preliminary recognition framework and not as a new diagnosis, for identifying patients who present with acute abdominal pain, preserved hemodynamics, and imaging findings disproportionate to clinical severity, while being supported by normal lactate levels. Its purpose is to help emergency physicians recognize patients who do not require surgery so that unnecessary surgical procedures can be avoided, with the definitive diagnostic workup proceeding in parallel. Corticosteroids may be considered on an individual basis when an immune-mediated etiology is reasonably suspected and competing infectious and surgical causes have been evaluated.

## Data Availability

The raw data supporting the conclusions of this article will be made available by the authors without undue reservation.
